# Identification of bladder cancer subtypes and predictive signature for prognosis, immune features, and immunotherapy based on immune checkpoint genes

**DOI:** 10.1038/s41598-024-65198-8

**Published:** 2024-06-23

**Authors:** Jiyue Wu, Feilong Zhang, Xiang Zheng, Dongshan Chen, Zhen Li, Qing Bi, Xuemeng Qiu, Zejia Sun, Wei Wang

**Affiliations:** Department of Urology, Beijing Chaoyang Hospital, Capital Medical University, 8 Gong Ti Nan Road, Chaoyang District, Beijing, 100020 China

**Keywords:** Bladder cancer, Immune checkpoints, Prognosis, Immunotherapy, Individualized treatment, Cancer, Urological cancer, Bladder cancer

## Abstract

Immunotherapy based on immune checkpoint genes (ICGs) has recently made significant progress in the treatment of bladder cancer patients, but many patients still cannot benefit from it. In the present study, we aimed to perform a comprehensive analysis of ICGs in bladder cancer tissues with the aim of evaluating patient responsiveness to immunotherapy and prognosis. We scored ICGs in each BLCA patient from TCGA and GEO databases by using ssGSEA and selected genes that were significantly associated with ICGs scores by using the WCGNA algorithm. NMF clustering analysis was performed to identify different bladder cancer molecular subtypes based on the expression of ICGs-related genes. Based on the immune related genes differentially expressed among subgroups, we further constructed a novel stratified model containing nine genes by uni-COX regression, LASSO regression, SVM algorithm and multi-COX regression. The model and the nomogram constructed based on the model can accurately predict the prognosis of bladder cancer patients. Besides, the patients classified based on this model have large differences in sensitivity to immunotherapy and chemotherapy, which can provide a reference for individualized treatment of bladder cancer.

## Introduction

Bladder cancer (BLCA) is the most common malignancy of the urinary system, characterized by high morbidity and mortality. According to statistics, there were 573,000 new diagnosed cases and 213,000 deaths of bladder cancer worldwide in 2020, ranking tenth among all cancers^1^. According to the invasion depth of tumor, bladder cancer can be divided into non-muscle invasive bladder cancer (NMIBC) and muscle invasive bladder cancer (MIBC), of which NMIBC accounts for about 75%^2^. In recent years, despite the continuous improvement of surgical approaches, and the application of platinum-based neoadjuvant therapy and intravesical instillation of Bacillus Calmette Guerin (BCG) after operation have made significant progress in the treatment of bladder cancer, but the prognosis of BLCA patients is still far from satisfactory^3^. It has been reported that 40–80% of patients with NMIBC recur within 1 year after surgery, and 10–25% of these patients will progress to MIBC^4^. MIBC is prone to invade blood vessels, lymph nodes and develop distant metastases to form metastatic BLCA, and the five-year survival rates for muscle invasive MIBC and metastatic bladder cancer are estimated to be only 36–48% and 5–36%, respectively^5^. Therefore, it is urgent to develop effective and reliable prognostic and treatment strategies to improve the quality of life and long-term survival of BLCA patients.

With the development of gene sequencing technology, therapies for BLCA have been developed at the cellular and molecular levels. Studies have shown that BLCA has a relatively high total mutation spectrum compared to other types of tumors, and the number of neoantigens produced by it may also be higher. Therefore, administering immunotherapy to BLCA patients may be a potentially effective therapeutic measure^6^. Recently, the rapid development of immune checkpoint inhibitors (ICIs) targeting programmed cell death molecule 1 (PD-1)/programmed cell death molecule ligand 1 (PD-L1) and cytotoxic T-lymphocyte antigen 4 (CTLA4) has brought new light to the treatment of BLCA^7^. Studies have shown that the median survival time of BLCA patients receiving immunotherapy was on average 3 months longer than that of patients receiving chemotherapy^8^. To date, the Food and Drug Administration (FDA) has approved several kinds of ICIs for the treatment of advanced BLCA patients^9^. It is worth noting that although the efficacy of ICIs is significantly better than conventional platinum-based chemotherapy, it is estimated that only 1/5 of patients can benefit from the treatment of ICIs^10^. Therefore, it is of great clinical significance to evaluate the responsiveness of BLCA patients to immunotherapy in order to achieve optimal and personalized treatment to improve the prognosis of these patients.

In this study, the ICGs score of each BLCA patient form The Cancer Genome Atlas (TCGA) and Gene Expression Omnibus (GEO) databases was calculated by using single sample gene set enrichment analysis (ssGSEA), and the genes significantly associated with ICGs scores were obtained by the weighted correlation network analysis (WCGNA) algorithm. The non-negative Matrix Factorization (NMF) machine learning method was applied to divide BLCA samples into two subgroups with different clinical, prognostic and immune characteristics according to the expression of the ICGs score-related genes. Based on the differentially expressed immune-related genes among the two subgroups, uni-Cox, Least absolute shrinkage and selection operator (LASSO), Support vector machine-recursive feature elimination (SVM-RF) and multi-Cox algorithms were performed to construct a new stratification model with nine genes. This model and the nomogram based on the model can accurately predict the prognosis of BLCA patients in TCGA and GEO databases. Besides, BLCA patients classified based on this model have a large difference in the sensitivity of immunotherapy and chemotherapy. Finally, in vitro experiments were conducted on a hub gene (CTSE) in the model for further exploration.

## Methods and materials

### Data acquisition and processing

The RNA sequencing data, somatic mutation data, gene copy number, and corresponding clinical information of BLCA patients (N = 414) were obtained from TCGA data portal (https://tcga-data.nci.nih.gov/tcga/). The gene expression matrix and clinical information of BLCA patients in GSE13507^11,12^ (N = 165) and GSE32894^13^ (N = 224) microarray datasets were obtained from GEO database. The IMvigor210CoreBiology R package was used to obtain transcriptomic data and clinical information from patients (N = 348) with metastatic urothelial carcinoma who received immunotherapy to validate the efficiency of our model in predicting the efficacy of immunotherapy^14^. The 82 ICGs involved in the analysis of this study were summarised from previously published studies.

Samples with incomplete transcriptome data and clinical information in each dataset were excluded from further analysis. The RNA sequencing data in TCGA-BLCA dataset were converted into log2(TPM + 1) to maintain the comparability with microarray dataset. Using the “sva” R package to eliminate the batch effect of microarray datasets.

### ICGs scoring and co-expression network construction

According to the expression level of 82 ICGs in TCGA-BLCA, GSE13507 and GSE32894 datasets, the ICGs score of each sample was scored using the ssGSEA function in the “GSVA” package. WCGNA analysis was used to identify genes module with similar expression patterns and analyze the correlation between genes module and ICGs scores. A hierarchical clustering tree was established by dynamic hybrid cutting method to identify co-expressed genes module. Each leaf of the tree represented a gene, and genes with similar expression patterns gathered to form a branch of the tree, and each branch represented a genes module. Pearson correlation analysis was used to calculate the correlation between modular characteristic genes and ICGs scores.

### NMF algorithm

The NMF algorithm was used to cluster the BLCA samples in the TCGA based on the intersection genes from the WGCNA analysis. The “dark” criterion was chosen and iterated 100 times. The number of clusters was set from 2 to 10. The average profile width of the common membership matrix was determined using the R package "NMF". The minimum number of members for each subclass was set to 10. The stability of clusters obtained by NMF was reflected by the value of the co-occurrence correlation, which is between 0 and 1. The higher the value, the greater the stability of the clusters. Besides, the smaller the value of the residual sum of squares (RSS), which reflects the clustering performance of the model, the better the clustering performance of the model. The optimal number of clusters is determined by co-occurrence, dispersion and contour measurement. Through the above algorithm and the optimal number of clusters, the samples are divided into different subtypes.

### Microenvironment scoring and immune infiltration assessing

Microenvironment scoring was performed by the “ESTIMATE” R package, which contains the stromal score, immune score, evaluation score and tumor purity of each sample. The infiltration of 23 kinds of immune cells in each sample was assessed by the “GSVA” R package based on the expression of marker genes of the immune cells.

### Functional enrichment analysis

The “clusterProfiler” R package was used for gene ontology (GO) and Kyoto Encyclopedia of Genes and Genomes (KEGG) enrichment analysis. Gene Set Enrichment Analysis (GSEA) was used to compare significantly different biological processes between the low-risk and high-risk groups. Pathways with FDR < 0.25 and *p* < 0.05 were considered to be statistically enriched. The “GSVA” R package was used for ssGSEA analysis to evaluate the activity of specific biological pathways in each sample and spearman correlation test was used to calculate the correlation between risk scores and pathway activity. The reference gene sets included in the specific biological pathways were obtained from the Molecular Signatures Database (MSiDB).

### Sensitivity prediction of immunotherapy and chemotherapy

The tumor immune dysfunction and rejection (TIDE) score was used to predict the immune escape potential of cancer cells and BLCA patients response to immunotherapy, with higher TIDE scores representing greater tumor immune escape potential and lower likelihood of immunotherapy benefit. The immune appearance score (IPS) for BLCA patients were obtained from the Cancer Immunome Atlas (TCIA) database to predict their responsiveness to immunotherapy with PD-1 and CTLA4 blockers. Besides, the IMvigor210 immunotherapy dataset was used to validate the association between patient risk characteristics and immunotherapy benefits.

The “pRRophetic” R package was used to predict the sensitivity of BLCA patients to chemotherapy. Specifically, the IC50 values of chemotherapeutic drugs were estimated from the gene expression profiles of BLCA samples by tenfold cross-validated ridge regression, using the gene expression profiles and IC50 values of cancer cells in the Genomics of Drug Sensitivity in Cancer (GDSC) database for the corresponding drug treatment scenarios as a reference.

### Tumor mutation load (TMB) calculation

TMB represents the number of mutations per million bases in tumor tissue, including genetic coding errors, base substitutions, base insertions and deletions. In theory, tumor cells with higher TMB may own high levels of neoantigens, which were believed to help the immune system recognize tumor cells and stimulate the proliferation and anti-tumor response of anti-tumor T cells. Therefore, immunotherapy against patients with higher TMB may be more effective. The TMB score of each patient was calculated based on somatic mutation data obtained from the TCGA database.

### Construction of a prognostic hierarchical model related to ICGs in BLCA patients

A differential expression gene analysis was performed on the different subtypes of BLCA obtained from the NMF algorithm, with |log2FC| > 1 and FDR < 0.05 set as thresholds. The TCGA dataset was randomly divided into a training set and a validation set in the ratio of 7:3. In the training set, genes with good prognostic ability (HR ≠ 1 and *p* value < 0.05) were identified by uni-COX regression analysis. To further reduce the number of candidate genes, the LASSO regression algorithm in the “glmnet” R package was used to remove overfitting bias by tenfold cross-validation to obtain a more concise prognostic gene set. Besides, feature genes closely related to BLCA patient survival were selected for survival features using the svm function of the “e1070” R package. SVM-RFE is a feature selection technology based on support vector machine, which sorts features according to the recursive feature deletion sequence. Ultimately, we select the number of feature genes holding the smallest value of the classification error. The results obtained from the above two algorithms were intersected to obtain candidate prognostic genes and a final model was constructed based on the above candidate prognostic genes by multi-COX regression. Coefficient values were derived for each gene and the risk score was equal to the expression of each gene multiplied by the corresponding regression coefficient.

$$ Risk\;score = \sum\limits_{i = 1}^{n} {\left( {{\text{coef}}_{i} \times {\text{Exp}}_{i} } \right)} $$

Time-dependent receiver operating characteristic (ROC) curve was used to assess the predictive power of the above risk models. Kaplan–Meier (K–M) curves were used to compare survival differences between patients in different risk groups. The accuracy of the risk models was also validated in the GSE13507 and GSE32894 datasets using the same methods.

### Independent prognostic analysis and construction of nomogram

To assess whether risk scores were independent prognostic factors for BLCA patients, uni-COX and multi-COX regression analyses were performed to screen for independent prognostic factors. The prognostic efficacy of each independent prognostic factor was assessed using c-index, time-dependent ROC curves and decision curve analysis (DCA). Finally, a nomogram containing clinical information and risk scores of BLCA patients was constructed using the “regplot” R package and calibration curves were drawn to assess the prognostic accuracy of the nomogram.

### Cell culture

Human bladder transitional cell carcinoma cells, T24 and J82 were purchased from American Type Culture Collection (ATCC). The culture method is as described previously. In brief, the cells were cultured in Dulbecco’s Modified Eagle Medium (DMEM) (D0822, Sigma-Aldrich, USA) containing 10% fetal bovine serum (FBS, F8687-500ML, Sigma-Aldrich, USA), 100 U/mL penicillin, and 100 U/mL streptomycin (PS, V900929-100ML, sigma-VETEC, USA). They were cultured at 37 °C in a humidified atmosphere with 5% CO_2_.

### Transfectin of CTSE adenovirus vector

The overexpression vector for CTSE and the negative control (pcDNA) were bought from Hanheng Biotehnology (Hanheng Biotehnology Co., Ltd., Shanghai, China). 80–90% confluenced cells were transfected with the Ad-CTSE for 24 h at least, the adenovirus vector was used at a 1:1000 dilution with DMEM.

### Real‑time quantitative PCR (RT‑qPCR)

Total RNA was isolated with TRIZOL Reagent (Invitrogen, 15596026) and reverse transcribed using a PrimeScript RT Master Mix kit (Takara, Japan) according to the manufacturer’s protocol. mRNA expression was determined by RT-PCR. The 2^−ΔΔCT^ method was used to quantify the mRNA expression. The primer sequences of CTSE and GAPDH in this study are listed below:

CTSE Forward: 5′-CATACAGCCAGCCAGGTCAA-3′

CTSE Reverse: 5′-GCTCCAATGATCCCGGACAA-3′

GAPDH Forward: 5′-GGTTGTCTCCTGCGACTTCA-3′

GAPDH Reverse: 5′-GGTGGTCCAGGGTTTCTTACTC-3′

### Cell proliferation assay

T24 cells and J 82 cells were seeded in 96-well plates at 1000 cells per well seperately. Cell proliferation was examined using the Cell Counting kit-8 (CCK-8, Ncmbio, China) following the manufacturer’s protocol. In brief, cells were collected at different time points and a 10 μL CCK-8 solution was added to each well. After incubation at 37 °C for 1 h, the light absorbance of each sample was measured at 450 nm.

### Colony formation assay

T24 cells and J82 cells were seeded in 6-well plates at 500 cells per well seperately, they were kept at 37 °C for 7 days to colonies formation, the medium changed every 72 h. Cells were washed and colonies were fixed using 4% paraformaldehyde (PFA) for 15–20 min after colony formation. The colonies were further stained with crystal violet for 25–30 min. Colonies were washed with PBS to remove additional staing and potographed.

### Statistical analysis

All statistical analyses involved in this study were performed by R software (version 4.1.0). Comparisons of continuous variables were done using the Student's t-test and comparisons of non-normally distributed variables were done using the Wilcoxon rank sum test. Comparisons of two sets of categorical variables were done using the chi-square test. All statistical tests were two-sided and *p* values < 0.05 were considered to be statistically different.

## Results

### Identification and enrichment analysis of gene modules associated with ICGs scores in BLCA

**Figure 1:**
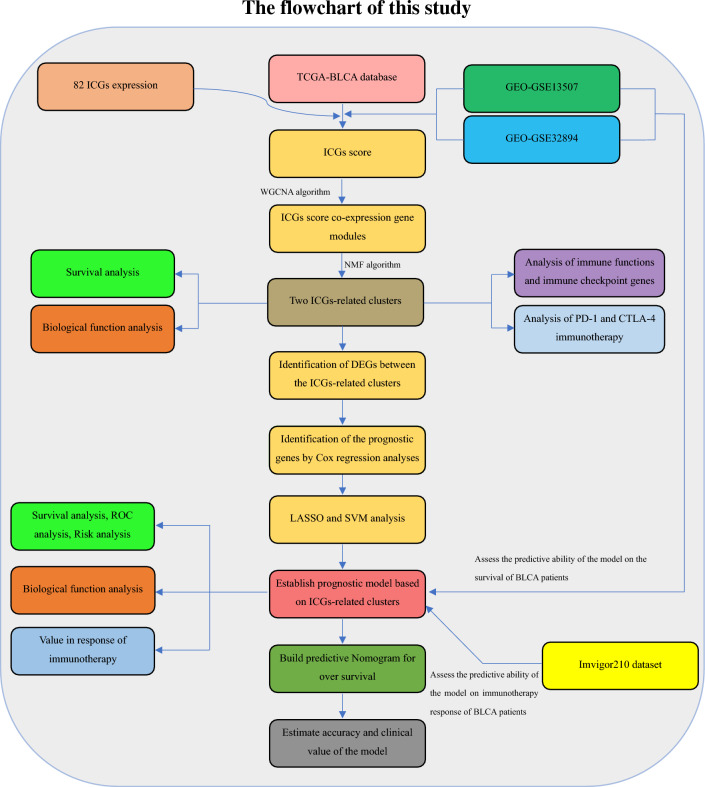
The flowchart of this study.

Figure 1 shows the flow chart for this study. The ICGs used for ssGSEA analysis are listed in Supplementary Table S1. The relationship between ICGs score and the age, gender, subtype, pathological grade and clinical stage of patients in TCGA-BLCA cohort was shown in Supplementary Figure S1. It can be seen that the ICGs score of female is higher than that of male patients. Besides, the higher ICGs score was likely to accompanied with the higher tumor malignancy (non-papillary, high grade and late stage). This indicated that ICGs score was associated with malignant progression of bladder cancer.

**Figure 2:**
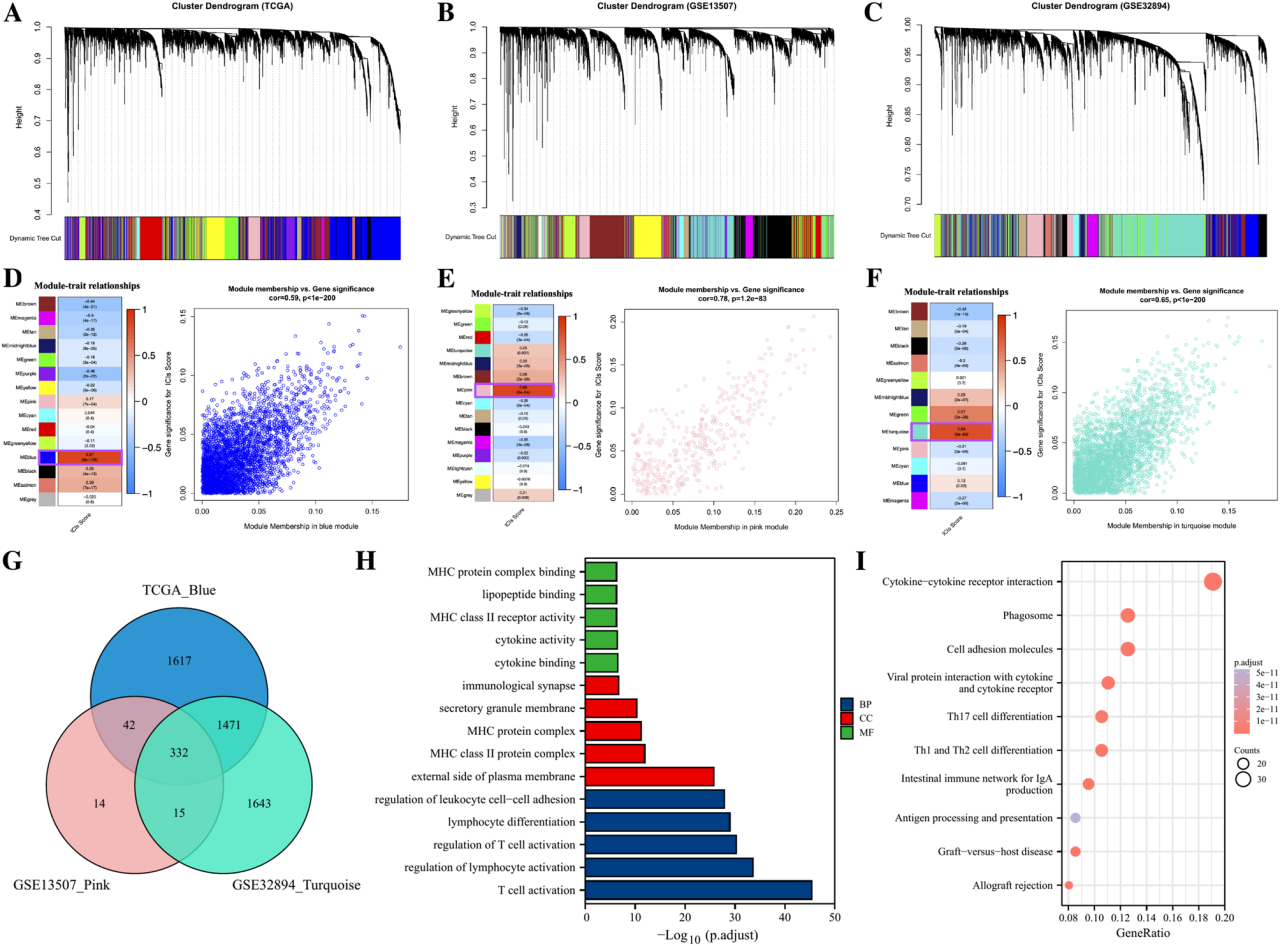
The co-expression network established using WGCNA. The WGCNA analysis of the (**A**) TCGA-BLCA database, (**B**) GSE13507 dataset, and (**C**) GSE32894 dataset. Heatmaps and scatter plots demonstrating the correlation between module eigengenes and ICGs scores in the (**D**) TCGA-BLCA dataset, (**E**) GSE13507 dataset, and (**F**) GSE32894 dataset. (**G**) Venn diagram displaying the ICGs score-related genes intersecting from different datasets. (**H**, **I**) Gene ontology (GO) and Kyoto Encyclopedia of Genes and Genomes (KEGG) analyses of ICGs score-related intersecting genes.

To further screen the gene module significantly associated with ICGs score, the WCGNA analysis showed that the blue module was highly correlated with the ICGs score among 15 modules in the TCGA cohort (R^2^ = 0.87, *p* = 8e − 126, Fig. 2A,D), the pink module was highly correlated with the ICGs score among 15 modules in GSE13507 cohort (R^2^ = 0.88, *p* = 6e − 54, Fig. 2B,E), the turquoise module was highly correlated with ICGs score among the 12 modules in the GSE32894 cohort (R^2^ = 0.84, *p* = 6e − 83, Fig. 2C,F). A total of 332 ICGs score-associated genes was identified by intersecting the 3 gene modules mentioned above (Fig. 2G). The results of GO enrichment analysis showed that these ICGs score-associated genes were mainly enriched in immune related biological processes such as T cell activation, regulation of lymphocyte activation, and lymphocyte differentiation (Fig. 2H). Besides, the results of KEGG pathway enrichment analysis were also enriched in immune related pathways such as Cytokine-cytokine receptor interaction, Th17 cell differentiation, Th1 and Th2 cell differentiation, Intestinal immune network for IgA production, and Antigen processing and presentation (Fig. 2I).

### Clinical characteristics of ICGs score-associated clusters

**Figure 3:**
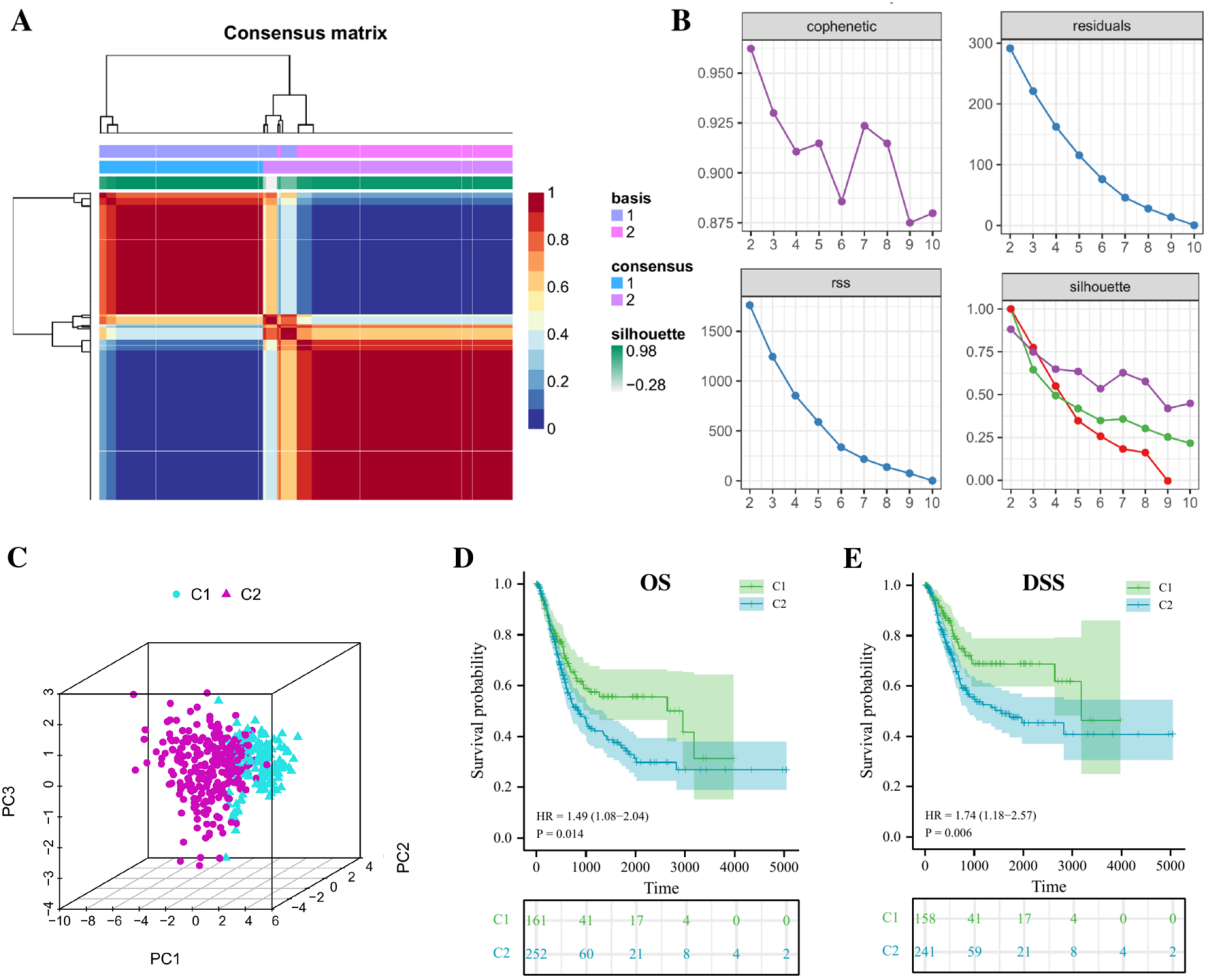
Molecular subtypes of BLCA based on ICGs score-related genes. (**A**) Consensus map of NMF clustering when k = 2. (**B**) Distribution of cophenetic, residuals, RSS and silhouette with a rank of 2–10. (**C**) A PCA plot of the expression profile of ICGs score-related genes between two clusters. (**D**, **E**) Prognostic survival curve of BLCA in molecular subtypes. ns, not significant.

Based on the above ICGs score-associated genes, TCGA-BLCA patients were clustered by NMF algorithm, and the optimal number of clusters was selected as 2 according to the steepness of the "cophenetic" decline (Fig. 3A,B). The PCA analysis showed that ICGs score-associated genes expression profile of the 161 patients in C1 subgroup were significantly different from that of the 253 patients in C2 subgroup (Fig. 3C). K–M prognostic analysis of the two subtypes showed that BLCA patients in C2 group had significantly worse OS (HR = 1.49, *p* = 0.014) and DSS (HR = 1.74, *p* = 0.006) than C1 group (Fig. 3D,E). The heatmap results of clinical pathological characteristics showed that some clinical characteristics of BLCA patients in C1 and C2 groups were also different, including histological subtype, pathological grade, clinical stage, tumor T stage and N stage (Supplementary Figure S2A).

### Biological characteristics of ICGs score-associated clusters

**Figure 4:**
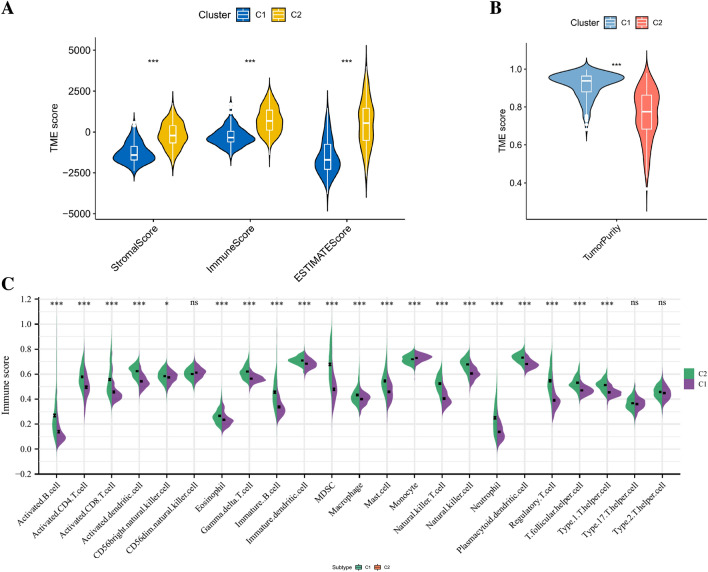
(**A**) The comparisons of stromal score, immune score, and estimate score between different clusters. (**B**) The comparisons of tumor purity between different clusters. (**C**) The Pod-plot illustrating the difference in immune cell infiltration between different clusters. ns, not significant; *p < 0.05; ***p < 0.001.

**Figure 5:**
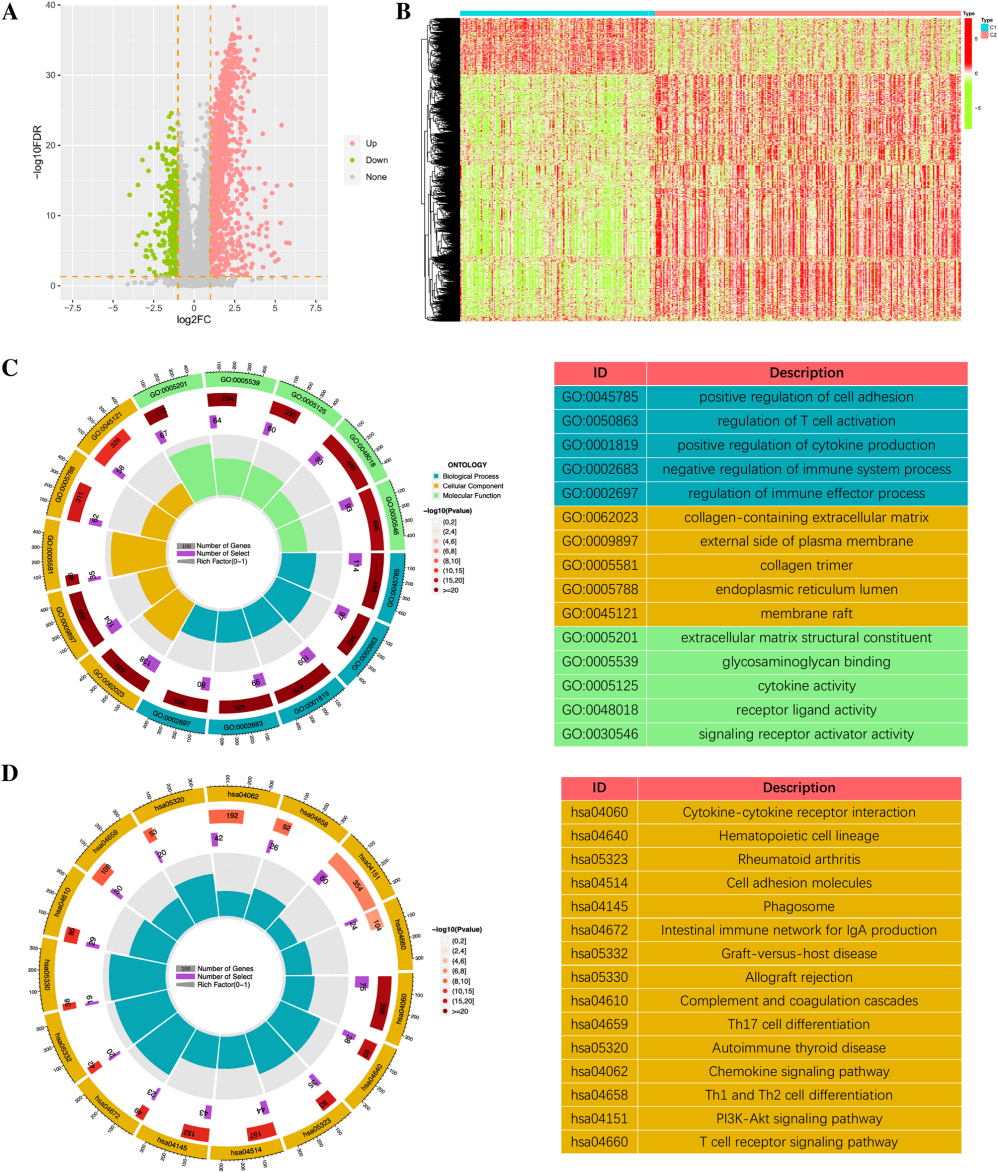
Screening and functional enrichment analysis of ICGs-related DEGs. (**A**) Volcano map showed the DEGs between different clusters. (**B**) Heatmap showing the DEGs between the two clusters. (**C**) GO analysis of the DEGs. (**D**) KEGG pathway analysis of the DEGs.

To verify the effectiveness of the above clustering methods, we compared the tumor microenvironment and immune cell infiltration of the two subgroups. As for tumor microenvironment analysis, the stromal score, immune score and estimate score were significantly higher in C2 group than in C1 group, while the tumor purity of C2 group was significantly lower than that of C1 group (Fig. 4A,B). The results of immune cell infiltration confirmed that the abundance of multiple immune cells including B cells, CD4T cells, CD8T cells, NK T cells, T regulatory cells, and macrophages were significantly higher in C2 group (Fig. 4C). Besides, we also compared the expression levels of 82 ICGs between the two BLCA subgroups, and the differentially expressed ICGs were most highly expressed in C2 group as shown in Supplementary Figure S2B. IPS analysis can be used to predict tumor response to ICIs. As shown in Supplementary Figure S2C–F, the IPS scores showed that the percentage of CLTA4 and PD1 positive cells was higher in C2 group than in C1 group while the percentage of CLTA4 and PD1 negative cells was lower in C2 group, indicating that the patients in C2 group had stronger immunogenicity, so immunotherapy may be more beneficial for patients in C2 group. To further investigate the potential mechanisms about differences between the two subgroups, the differential analysis of the gene expression profiles between the two subgroups were performed and 1371 differentially expressed genes (DEGs) were obtained. The volcano plot of difference analysis and the heatmap of DEGs between two subgroups were shown in Fig. 5A,B. Subsequently, the GO and KEGG enrichment analysis on these 1371 DEGs were performed. The results of GO enrichment analysis were mainly focused on immune-related biological processes such as immune cell activation, cytokine production, and regulation of immune effector process (Fig. 5C). The results of KEGG pathway enrichment analysis also included many pathways associated with immune regulation such as cytokine-cytokine receptor interaction, intestinal immune network for IgA production, Th17 cell differentiation, chemokine signaling pathway, PI3K-Akt signaling pathway, T cell receptor signaling pathway, etc. (Fig. 5D). All the above results indicated that there are great differences in tumor immunity between the two subgroups classified by NMF algorithm.

### Construction and validation of Immune-related model

**Figure 6:**
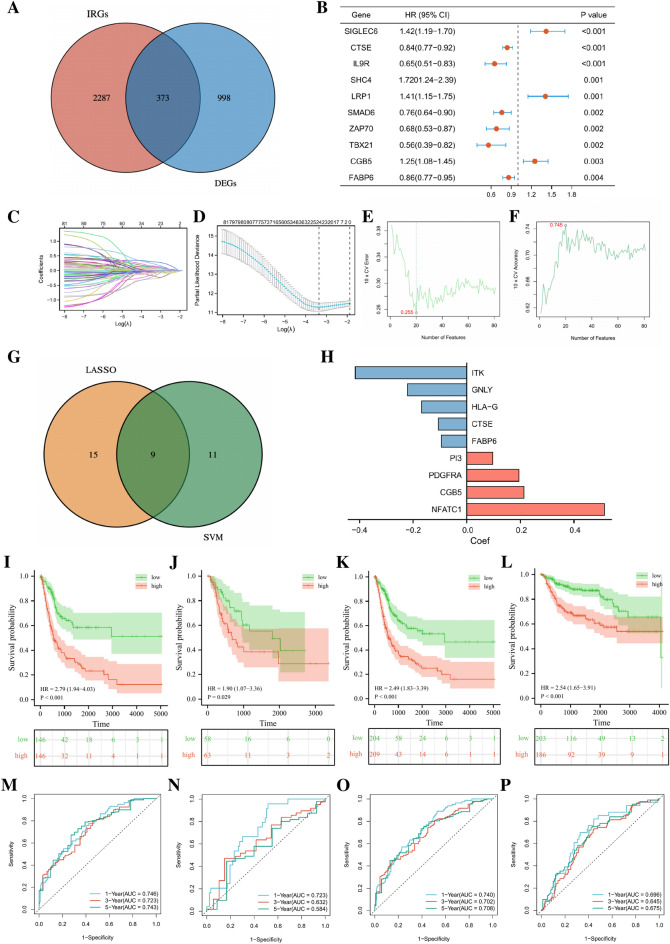
Construction of a prognostic model based on ICGs score related genes. (**A**) The Venn diagram shows the process of intersection between IRGs and DEGs. (**B**) Forest plot shows the top ten prognostic genes in the training set. (**C**, **D**) LASSO regression for further screening of prognosis-related genes, with the optimal log(Lambda) when n = 24. (**E**, **F**) SVM screening of prognosis-associated genes, with the highest accuracy achieved at n = 20 after tenfold cross validation. (**G**) The genes further screened by LASSO were intersected with those obtained by SVM to obtain candidate genes. (**H**) Regression coefficients obtained by multivariate cox regression analysis of candidate genes. The K–M curve shows the OS of high-risk and low-risk patients in different data sets. (**I**) TCGA training set. (**J**) TCGA validation set. (**K**) TCGA overall set. (**L**) GSE13507 and GSE32894 datasets. ROC curves show the prognostic value of the constructed model in different datasets. (**M**) TCGA training set, (**N**) TCGA validation set. (**O**) TCGA overall set. (**P**) GSE13507 and GSE32894 datasets.

To better classify the above subgroups for clinical treatment guidance and quantify the specific risk score of each BLCA patient. A total of 373 immune-related differentially expressed genes (IR-DEGs) related to immunity were obtained by intersecting 1371 DEGs with immune-related genes (IRGs) from the Immport database (a database for collecting, organizing, and sharing immunology related research resources, https://www.immport.org/home) (Fig. 6A). Firstly, uni-COX regression was performed on the above IR-DEGs, and 81 genes with significant prognostic value were identified in the training set (Fig. 6B). Then, LASSO regression analysis was used to further screen the 81 genes to simplify the final risk model (Fig. 6C,D). Meanwhile, the best classification model based on the survival status of BLCA patients was identified by SVM-RFE algorithm, and 20 characteristic genes were selected corresponding to the smallest classification error (0.255) (Fig. 6E,F). Finally, 9 candidate genes were confirmed by integrating the results from the LASSO regression and SVM analysis (Fig. 6G), which were subjected to multi-COX regression analysis to construct the final model. The risk score (RS) of each patient is the expression of 9 candidate genes multiplied by the coefficient of their multi-COX regression (Fig. 6H). According to the median value of RS in the training set, the BLCA patients were divided into low and high risk groups in TCGA training set, TCGA validation set, overall TCGA set, external validation set GSE3507 and GSE32894. The results of the K–M curve survival analysis showed that no matter in which cohort, the OS in high risk group was significantly worse than that in low risk group (Fig. 6I–L). In TCGA training set, the AUCs for 1y, 3y and 5y OS were 0.75, 0.72 and 0.74, respectively; for TCGA training set, the AUCs for 1y, 3y and 5y OS were 0.72, 0.63 and 0.58; for overall TCGA set, the AUCs for 1y, 3y and 5y OS were 0.74, 0.70 and 0.71; for the external validation set, the AUCs for 1y, 3y and 5y OS were 0.70, 0.65 and 0.68 (Fig. 6M–P). Besides, the results of K–M analysis in different TCGA subgroups (including age, gender, BMI and clinical stage of the tumor) also showed that patients in high risk group had a worse prognosis than those in low risk group (Supplementary Figure S3A–D). The above results all indicated that the risk model we constructed had strong predictive efficacy. In general, MIBC patients among BLCA usually have worse prognosis. Accurately predicting the prognostic outcome of these BLCA patients is of great significance for early clinical intervention and active treatment. Therefore, we conducted a K–M survival analysis on MIBC patients using the model constructed above in the TCGA training set, the TCGA validation set, the overall TCGA set and GEO external validation set. The results showed that for MIBC patients, high-risk patients classified by our model also had worse prognosis (Supplementary Figure S4A,B). It can be seen that our prognostic model also has high accuracy for MIBC patients.

### Correlation analysis of clinical characteristics and construction of nomogram

**Figure 7:**
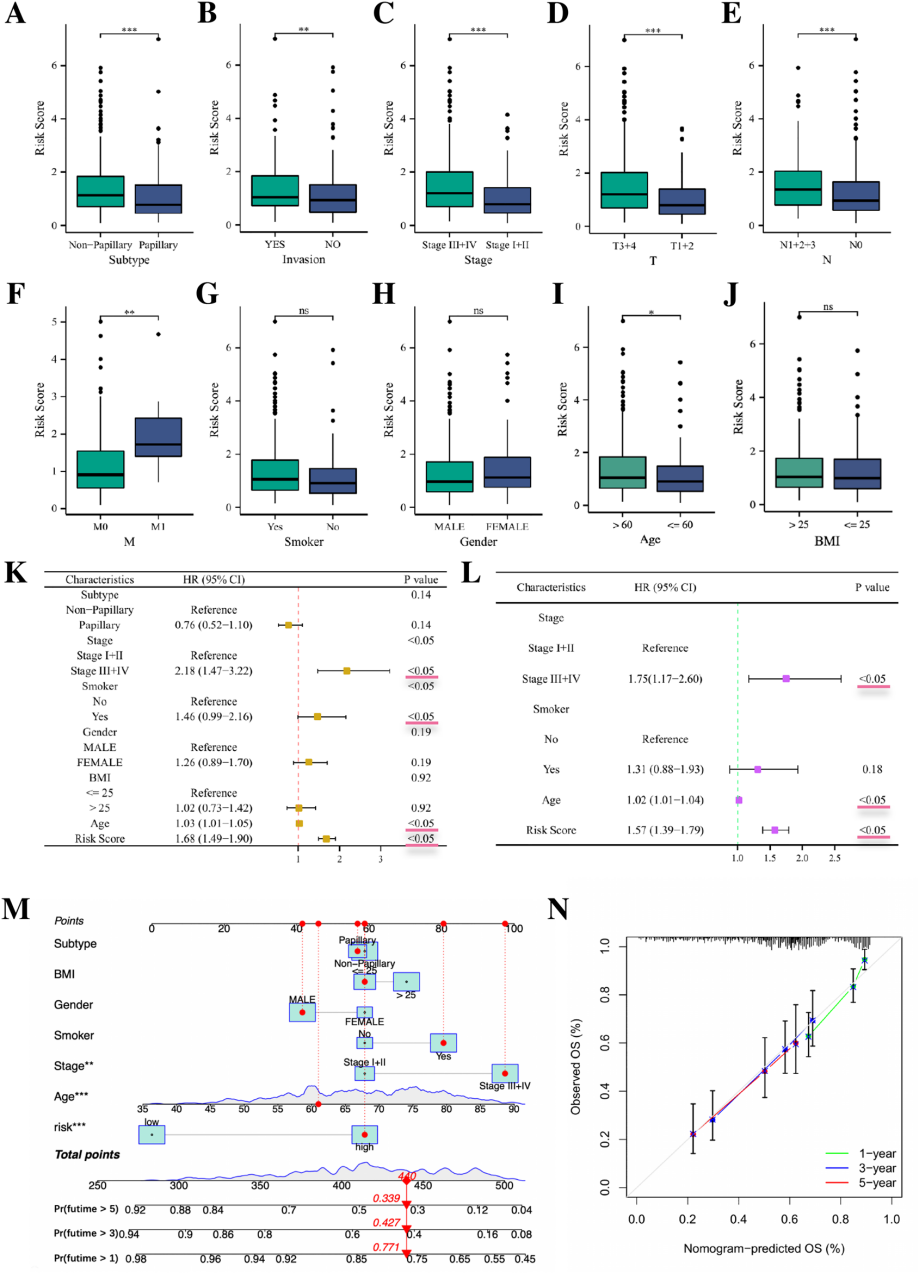
Clinical correlation analysis. (**A**–**J**) box plot shows the correlation between patient risk scores and clinical parameters. (**K**) Forest plot shows uni-COX regression analysis of BLCA patients. (**L**) Forest plot shows the multi-COX regression analysis of BLCA patients. (**M**) Prognostic line plot constructed by integrating multiple clinical parameters of patients. (**N**) Calibration plots of the nomogram at 1, 3 and 5 years.

The results of the clinical correlation analysis showed that patients with non-papillary tumors, presence of lymph-vascular invasion, late clinical stage, higher TNM stage and older age had higher risk scores, whereas clinical characteristics such as whether the patient smoked, patient gender and BMI did not seem to be related to the risk scores (Fig. 7B–J). In order to determine whether the risk score was an independent prognostic factor for BLCA patients, we performed uni-COX and multi-COX analyses, and the results confirmed that the risk model based on 9-gene expression was an independent prognostic factor for BLCA patients. Besides, the patient’s age and clinical stage of the tumor were also found to be independent prognostic factors for BLCA patients (Fig. 7K,L). The results of the C-index, ROC curve and DCA curve analyses all showed better predictive power of the risk score compared to patient’s age and tumor clinical stage (Supplementary Figure S5). To provide a more powerful clinical prediction scheme, a prognostic nomogram was constructed by integrating multiple clinical information and risk scores, which intuitively showed the estimated survival of BLCA patients at 1, 3 and 5 years (Fig. 7M). The calibration chart of the nomogram showed good agreement between the survival rates predicted by the nomogram and the actual survival rates (Fig. 7N), indicating that the nomogram we constructed has good prognostic value for BLCA patients.

### Enrichment analysis based on risk model

**Figure 8:**
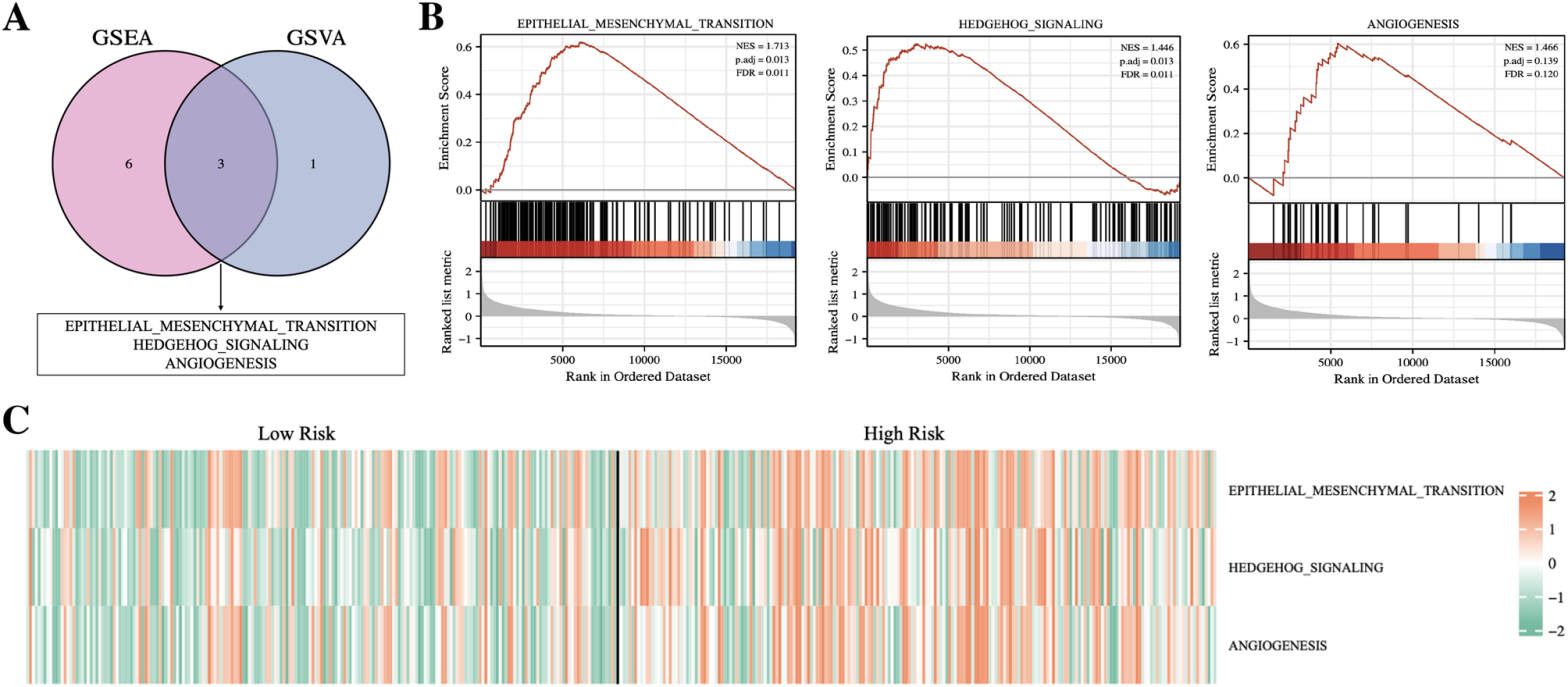
Function enrichment analysis. (**A**) Venn diagram shows the results of GSVA and GSEA enrichment analysis. (**B**) The enrichment results of EMT, hedgehog signaling pathway and angiogenic signaling pathway in GSEA analysis. (**C**) Heat map shows the enrichment levels of the three signaling pathways in different samples in GSVA analysis.

To further explore the potential biological mechanisms that leading to the differences between the two groups (high risk and low risk), we performed GSEA and GSVA enrichment analysis based on the Hallmarks gene set (h.all.v7.2.symbols.gmt) in the MSigDB database. The results of both types of enrichment analysis showed that three distinct characteristics including EMT, hedgehog signaling pathway activation and angiogenesis were evident in the high-risk group compared to the low-risk group (Fig. 8A). The results of the GSEA analysis for the three characteristics were shown in Fig. 8B, and a heatmap of the GSVA enrichment scores for the three characteristics of each patient was shown in Fig. 8C.

### Guidance of risk model for immunotherapy in BLCA patients

**Figure 9:**
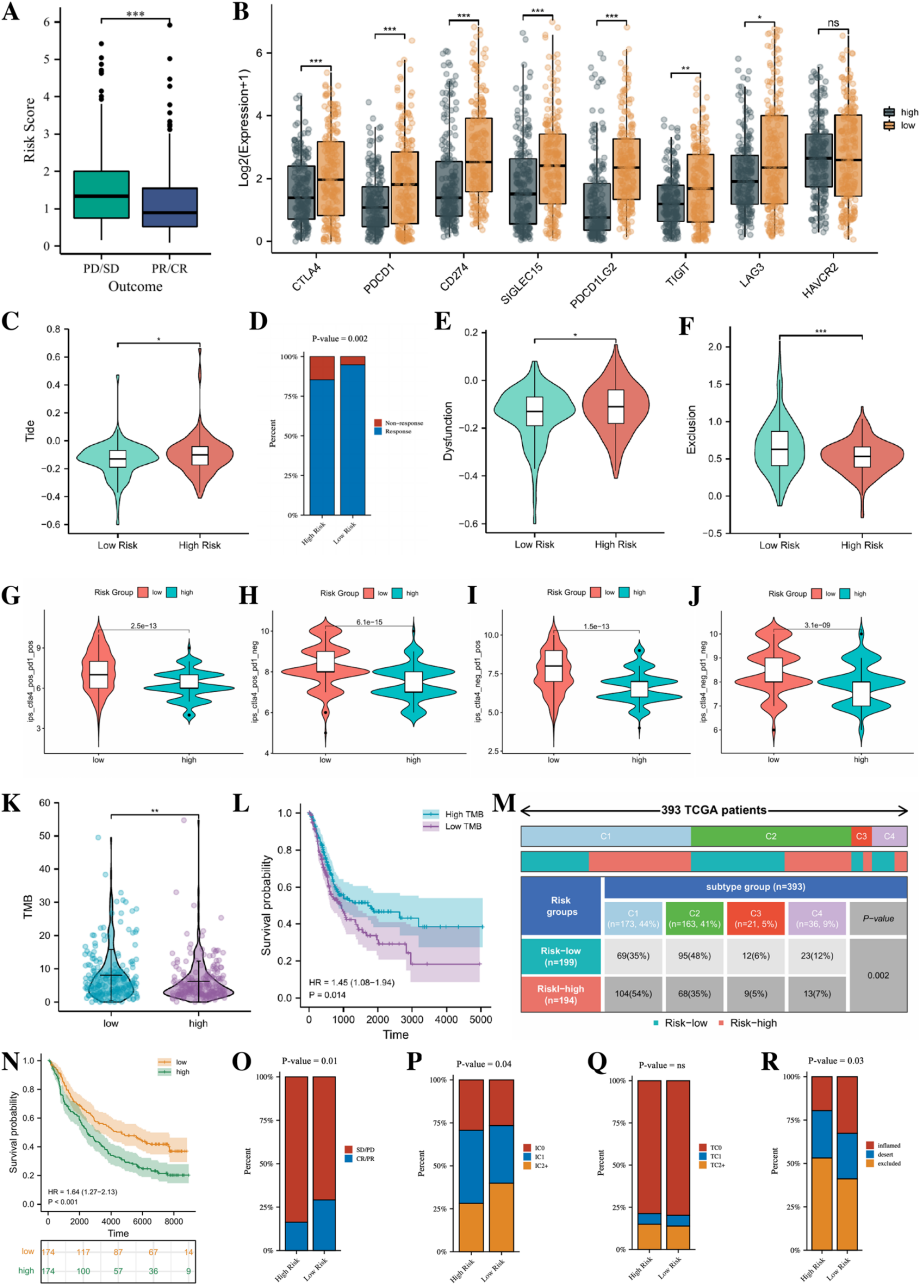
Sensitivity analysis of immunotherapy. (**A**) Comparison of treatment benefits in patients with different risks. (**B**) Expression of eight classical immune checkpoint genes in patients with different risks. (**C**) TIDE scoring of patients with different risks. (**D**) Percentage of immune response of BLCA patients in different risk groups. (**E**, **F**) Dysfunction and exclusion scores of patients with different risks. The comparison of immunophenoscore (IPS) between different risk groups. (**G**) CTLA4−_ PD1−. (**H**) CTLA4−_ PD1+. (**I**) CTLA4+_ PD1− and (**J**) CTLA4+_ PD1+. ns, not significant. (**K**) TMB of patients with different risks. (**L**) K–M curve shows the OS difference of patients with high and low TMB. (**M**) Comparison of the differences in immune subtype between different risk groups. (**N**) The Kaplan–Meier curve survival analysis between the high- and low- risk groups in the IMvigor210 cohort. Percentage of CR/PR and SD/PD (**O**) immune cell (IC) level type (**P**) tumor cell (TC) level type (**Q**) and immune subtypes (**R**) among risk groups of BLCA patients.

In the TCGA cohort, 357 patients received one or more therapeutic measures (such as BCG perfusion, chemotherapy such as cisplatin, gemcitabine, etc., uurgical treatment such as TURBT), including 256 patients with CR/PR and 101 patients with PD/SD. Comparison of risk scores between the two groups revealed that patients with PD/SD had significantly higher risk scores than patients with CR/PR (Fig. 9A). In order to better provide individualized treatment guidance for BLCA patients, the role of risk score in guidance of immunotherapy was comprehensively analyzed. As shown in Fig. 9B, some classical immune checkpoint molecules including CD274, CTLA4, PDCD1 and SIGLEC15, were more highly expressed in low-risk patients, which seems to suggest that these patients would benefit more from the administration of the corresponding ICIs. Based on the TIDE algorithm, we observed higher Tide scores, Dysfunction scores and lower Exclusion scores in high-risk patients compared to low-risk patients. This indicated that the lower the possibility of immune escape of low-risk patients and the more effective the immunotherapy may be on these patients (Fig. 9C,E,F). For predicting immunotherapy response, we found that patients in the low-risk group may have a stronger response to immunotherapy compared to those in the high-risk group (Fig. 9D). For IPS scores, we found that all four IPS scores were higher in low-risk patients than that in high-risk patients, which further indicated that low-risk patients may be more sensitive to immunotherapy (Fig. 9G–J). We also analyzed the relationship between patient risk scores and TMB, which is considered to be another potential indicator for assessing immunotherapy. It has been reported that the higher TMB the tumor cells hold, the more neoantigens it is exposed and therefore it is more likely to be recognized and cleared by the immune system. As shown in Fig. 9K, the TMB was higher in low-risk patients than that in high-risk patients, which further confirmed that patients in the low-risk group may be more sensitive to immunotherapy. Besides, the results of the K–M analysis also indicated that survival was also worse in patients with low TMB than in those with high TMB (Fig. 9L). Figure 9M showed the ratio of TCGA immune subtypes^15^ (C1 means Wound Healing Subtype, C2 means IFN-gamma Dominant Subtype, C3 means Inflammatory Subtype, C4 means Lymphocyte Depleted) between the different risk groups, and the results suggested that there were indeed significant differences in the immune phenotypes of patients in the different risk groups.

We further verified the patients’ response to immunotherapy in the IMvigor210 cohort. Figure 9N showed that the risk score calculated based on the 9-gene model divided patients into high and low risk group. And consistent with TCGA and GEO cohorts, the OS of high-risk patients in IMvigor210 cohort was worse than that of low-risk patients. The analysis of the immunotherapy showed that patients in the low-risk group responded better to immunotherapy than those in the high-risk group (Fig. 9O). The percentage of immune cells expressing PD-L1 was higher in the low-risk group compared to the high-risk patients, while there was no significant difference in the percentage of tumor cells expressing PD-L1 (Fig. 9P,Q). And the results of the inflammatory immune subtype analysis showed that the low-risk patients had more “inflammation” tumors compared to the high-risk patients, while the high-risk patients had more “excluded” tumors compared to the low-risk patients (Fig. 9R).

### The guiding significance of risk model for chemotherapy in BLCA patients

**Figure 10:**
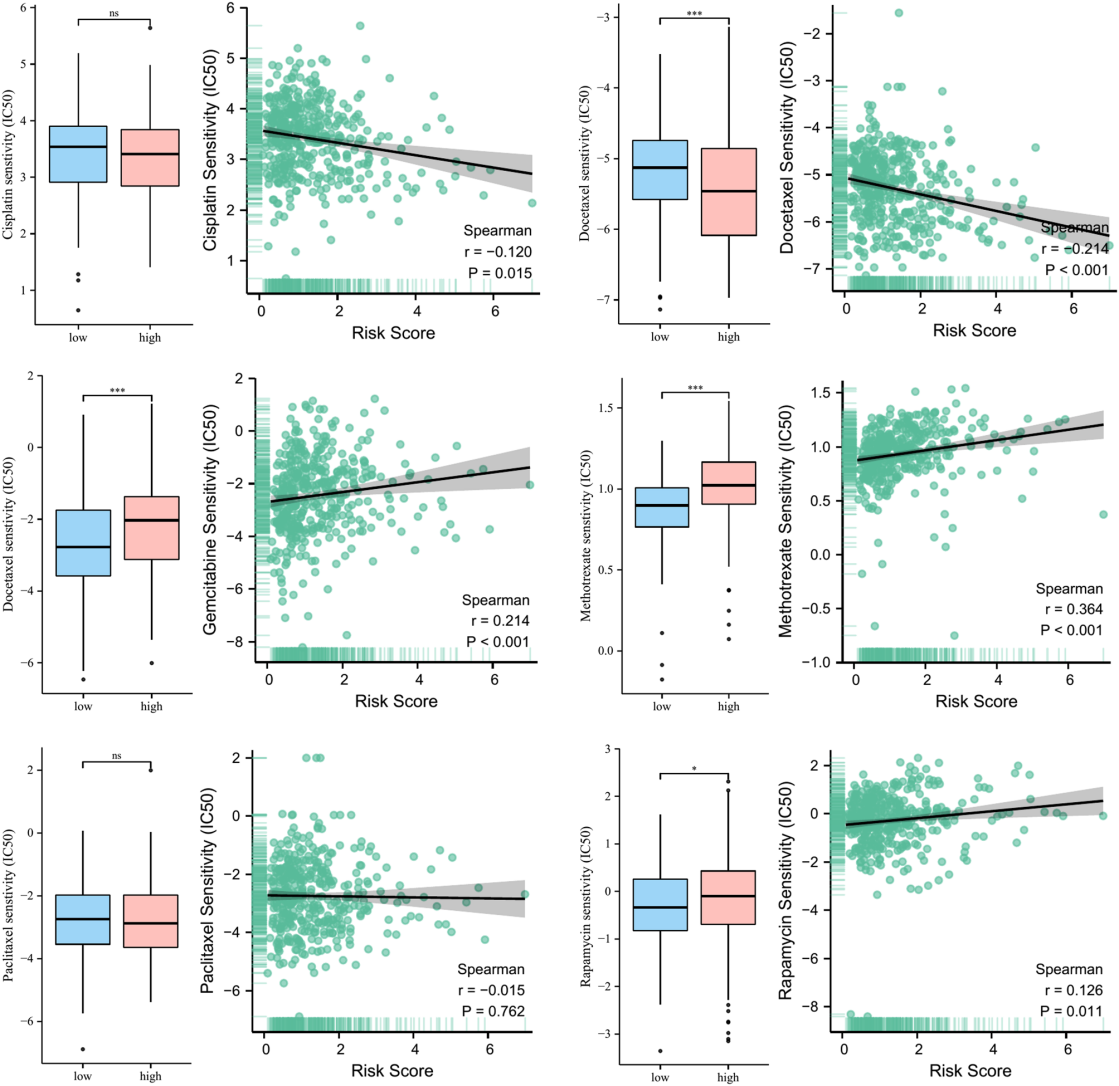
Drug sensitivity analysis.

To explore the guiding role of risk scores in chemotherapy of BLCA patients, we evaluated the IC50 values of risk scores against several common chemotherapeutic agents such as cisplatin, doxorubicin, gemcitabine, methotrexate, paclitaxel, and rapamycin. The results in Fig. 10 suggested that patients in the high-risk group may be more sensitive to doxorubicin, while patients in the low-risk group may benefit more from chemotherapy with gemcitabine, methotrexate and rapamycin.

### The gene CTSE in the risk model is closely related to the development of BLCA

**Figure 11:**
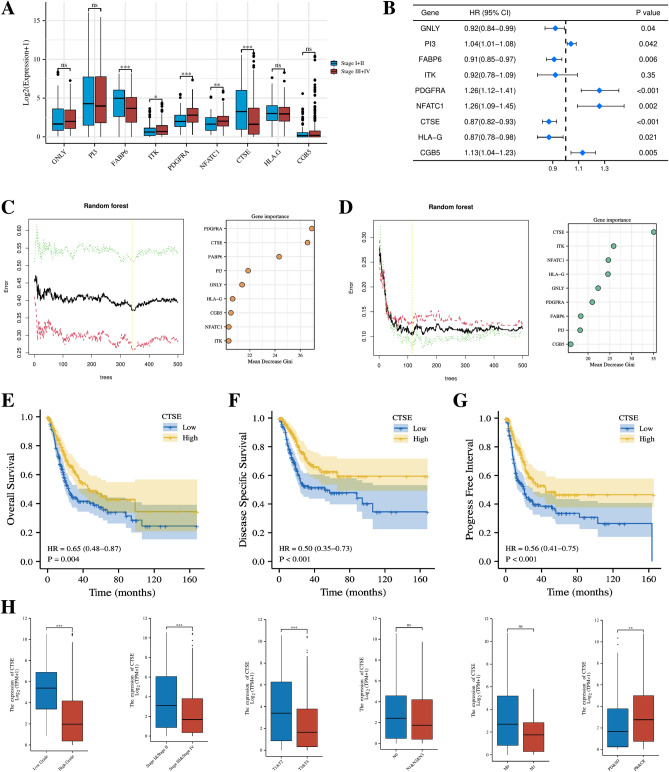
Selection of key genes for subsequent experimental validation. (**A**) Correlation between 9 genes and clinical analysis in BLCA patients. (**B**) uni-COX analysis of the 9 model genes. (**C**) Importance ranking of genes according to patient risk grouping by randomized forest algorithm. (**D**) Importance ranking of genes according to patient survival status by randomized forest algorithm. K–M curve shows differences in OS (**E**), DSS (**F**) and PFS (**G**) in patients with different CTSE expression. (**H**) Correlation analysis of CTSE expression levels with clinical parameters in BLCA patients.

For the nine genes in the risk model, boxplot showed that FABP6, ITK, PDGFRA, NFATC and CTSE were strongly correlated with the clinical stage of the BLCA patients (Fig. 11A). The results of uni-COX analysis showed that all the model genes excepting ITK, were closely associated with the prognosis of BLCA patients (Fig. 11B). To evaluate the importance of the prognostic contribution of the above genes to BLCA patients, we performed random forest algorithm on the nine genes based on risk scores and patient survival, and finally found that CTSE had the highest mean decrease gini in both analysis (Fig. 11C,D). Considering the important impact of CTSE on the survival and prognosis of BLCA patients, we selected CTSE for an in-depth study. Through K–M survival analysis, we found that BLCA patients with low CTSE expression had significantly worse OS, DSS and PFI than BLCA patients with high CTSE expression (Fig. 11E–G). The clinical correlation analysis showed that the expression level of CTSE was lower in BLCA patients with higher pathological grade, late clinical stage, late T stage and poor treatment, which further proved that the expression level of CTSE was closely related to the development of BLCA (Fig. 11H).

### CTSE inhibits the progression from NMBLC to MBLC

**Figure 12:**
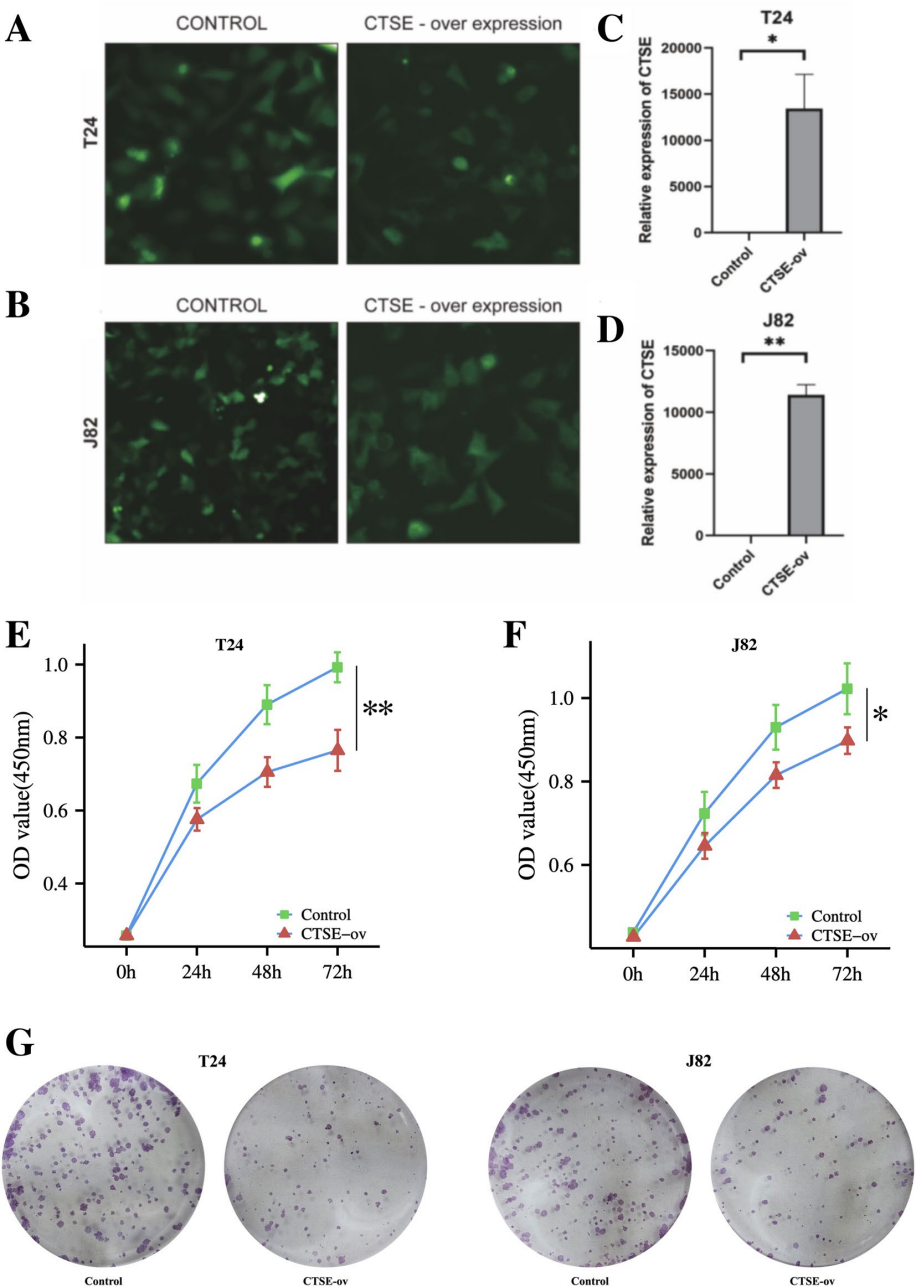
CTSE over expression inhibits the proliferation of T24cells and J82cells. (**A**) Transfection of Ad-CTSE adenovirus in T24 cells. (**B**) Transfection of Ad-CTSE adenovirus in J82 cells. (**C**) The relative expression level of CTSE were determined using qPCR in T24 cells. (**D**) The relative expression level of CTSE were determined using qPCR in J82 cells. (**E**) The results of CCK-8 assay in T24 cells. (**F**) The results of CCK-8 assay in and in J82 cells. (**G**) The colony formation in T24 cells and in J82 cells.

To clarify the relationship between CTSE gene and BLCA, the CTSE overexpression was constructed in two human bladder transitional cell carcinoma cells lines T24 and J82 by transfecting with Ad-CTSE adenovirus (Fig. 12A,B), and the expression level of CTSE in the transfected group increased significantly after the successful construction (Fig. 12C,D). The CCK-8 results showed that the cells activity of the CTSE over-expression in T24 cells and J82 cells were significantly lower than that of the control group (Fig. 12E,F). At the same time, the colony formation assay showed that the colony formation of the CTSE over-expression in T24 cells and J82 cells were significantly inhibited compared with the control group (Fig. 12G). These results all suggest that CTSE can inhibit the progression of tumor cells.

## Discussion

With the increasing in BLCA diagnosed, it has become a major burden for health care in the world. Given its great heterogeneity at the cellular, molecular, and genomic levels, BLCA patients differ greatly in many aspects such as clinical characteristics and therapeutic responsiveness^16,17^. Although significant progress has been made in the past decades with conventional treatment regimens, the survival rate and quality of life in BLCA patients have not improved significantly^18^. Immunotherapy is under extensive discussion in recent years, and immune checkpoint molecules are the key of immunotherapy. Immune checkpoint molecules are a kind of membrane protein that play a pivotal role in immune homeostasis by regulating the activation of immune cells^19^. Currently, several ICIs targeting immune checkpoints have shown great potential in the treatment of various malignancies including BLCA, and have been approved by the FDA for clinical application^20^. However, despite the higher efficacy and tolerance of ICIs have been confirmed in BLCA patients, there are still many patients cannot benefit from them^21^. It has been suggested that the expression levels of classical ICGs such as CTLA4, PD-1, and PD-L1 may not accurately identify patients who may benefit from immunotherapy, and a comprehensive analysis of ICGs in bladder tumor tissues may be able to more accurately assess the responsiveness of BLCA patients to immunotherapy^22^.

In this study, we collected as many ICGs as possible mentioned in the published literature. BLCA patients in TCGA and GEO datasets were scored for ICGs according to the expression of 82 ICGs, and genes closely related to ICGs scores were obtained by the WCGNA algorithm. Based on the ICGs scores-related genes, the BLCA patients can be divided into two subgroups with significant differences in prognosis, clinical and immune characteristics by the NMF algorithm. The results of survival analysis showed that the OS and DSS of patients in C2 subgroup were significantly worse than C1 subgroup. In the comparison of clinical characteristics, the tumors with non-papillary tumors, higher pathological grade, advanced clinical stage, deeper bladder wall invasion and more severe lymph node metastasis were observed more commonly in C2 subgroup. The results of tumor microenvironment and immune cell infiltration analysis showed higher scores of immune, stromal and higher infiltration abundance of immune cells such as CD4 + T cells, CD8 + T cells and B cells in C2 subgroup. Besides, for the ICGs differentially expressed between the two subtypes, their expression was significantly higher in C2 subgroup than in C1 subgroup, and the results of the IPS immunotherapy analysis also confirmed that the response to ICIs did appear to be better in C2 patients than in C1 patients. The results of enrichment analysis showed that the differentially expressed genes between the two subtypes were mainly concentrated in a variety of immune related biological processes and signaling pathways.

The BLCA patients were classified into high-risk and low-risk groups based on the differentially expressed immune-related genes between the two subtypes using uni-COX, LASSO, SVM algorithms and multi-COX. In the TCGA and GEO patient cohorts, regardless of whether it is NMIBC or MIBC subtype, the OS of high-risk patients is worse than that of low-risk patients. The clinical correlation analysis showed that the risk score was closely related to age, pathological subtype, vascular lymph invasion, pathological grade, clinical stage and TNM stage of BLCA. Independent prognostic analysis showed that age, clinical stage of tumor and risk score were independent risk factors for BLCA. The nomogram constructed by integrating the above risk factors was used to predict the prognosis of BLCA patients, and the results were highly consistent with the actual survival rate of BLCA patients. To facilitate the individualized treatment of BLCA patients, we evaluated the responsiveness of two types of patients to immunotherapy by TIDE algorithm, IPS score and TMB analysis, and the results all showed that low-risk patients seemed more likely to benefit from immunotherapy. The above analysis results were further verified through IMvigor210 dataset, which showed that patients in the low-risk group had better responsiveness to immunotherapy than those in the high-risk group. The analysis of immune subtypes showed that there were more “inflamed” tumors in low-risk patients than in high-risk patients, and fewer “excluded” tumors than in high-risk patients.

The results of GSEA and GSVA enrichment analysis showed three distinct features including epithelial mesenchymal transition, hedgehog signaling pathway activation and angiogenesis in high-risk patients compared to the low-risk patients. Epithelial mesenchymal transition is considered be an important feature in the malignant progression of bladder cancer, which endows the urothelial cells with reprogramming, loss of apical polarity, and gains the ability to self-renewal, immune escape, and metastasis^23^. Hedgehog signaling plays an important role in embryonic development, controlling cell proliferation and fate^24^. Studies have shown that activation of hedgehog signaling pathway promotes the survival and renewal of cancer stem cells (CSCs) in BLCA and enhances the tolerance of BLCA to various chemotherapeutic agents^25^. Besides, EMT may be induced by activating hedgehog signaling pathway at the early stage of tumor^26^. Tumor angiogenesis is a complex process, which can inhibit the apoptosis of BLCA cells, promote their proliferation and migration, and enhance their immune escape^27^.

For the nine genes in the model, FABP6, PDGFRA, NFATC1 and CTSE were found to be strongly associated with prognosis and clinical staging of BLCA patients^28^. Consistent with this study, Lin et al. identified FABP6 as a protective gene in BLCA, and patients with low expression of FABP6 have a better prognosis^29^. However, in another study, FABP6 was found to promote bladder cancer cell growth and cisplatin resistance. The specific reasons for these contradictory results are unclear so far, which deserves to be further explored in the future. PDGFRA has been widely explored in gastrointestinal tumors. Studies have shown that it can lead to the occurrence and progression of a variety of gastrointestinal tumors, and is considered as a potential target for anti-cancer treatment in gastric cancer^30^. In BLCA, Mezheyeuski et al. found that PDGFRA was significantly up-regulated in tissues, and its expression level in tissues was also significantly related to the prognosis of BLCA patients^31^. NFATC1 increase BLCA to promote tumor cell growth/colony formation, and can also promote tumor migration and invasion by providing the expression of MMP2 and MMP9. In addition, NFATC1 was significantly higher in tumor tissues than in benign urothelium. The high expression of NFATC1 was significantly associated with poor prognosis. NFATC1 increased BLCA to promote tumor cell growth/colony formation and also promoted tumor migration and invasion by increasing expression of MMP2 and MMP9. In addition, NFATC1 was significantly higher in tumor tissues than in benign uroepithelium, and high NFATC1 expression was significantly associated with poor prognosis^32^. CTSE was identified as the most critical gene in the model. In recent years, CTSE was found to be differentially expressed in a variety of tumor tissues and its expression level was closely associated with the occurrence and development of tumors^33^. For BLCA, in an earlier retrospective study, CTSE was considered as an independent prognostic marker for NMIBC and could be used to guide the treatment^34^. Blaveri et al. found that CTSE was overexpressed in NMIBC compared with MIBC. In a long-term follow-up study of 693 NMIBC cases, it was confirmed that low CTSE expression was significantly associated with the progression of NMIBC to MIBC^35^. Up to now, there are relatively few studies on the role of CTSE in the occurrence and development of BLCA. In our study, we found that overexpression of CTSE inhibited the proliferation and colony formation in different bladder cancer cell lines, which indicated the inhibition in tumor progression of CTSE.

In conclusion, in this study, we performed a comprehensive analysis of the expression profile of ICGs in BLCA tissues, and constructed an immune-related model based on the global landscape of ICGs. The high and low risk patients stratified by the model are different in many aspects such as prognosis prediction, clinical characteristics and treatment sensitivity. Early identification, timely intervention and individualized treatment of the two groups of patients based on the results of our model will help improve quality of life and long-term survival.

### Supplementary Information

Supplementary Information 1.

Supplementary Information 2.

Supplementary Information 3.

Supplementary Information 4.

Supplementary Information 5.

Supplementary Information 6.

## Data Availability

The datasets analysed during the current study are available in the TCGA database (TCGA-BLCA, https://portal.gdc.cancer.gov) and GEO database (GSE13507 and GSE32894, https://www.ncbi.nlm.nih.gov/geo/). Further inquiries can be directed to the corresponding author.
