# Supplementary material for: Identification of bladder cancer subtypes and predictive signature for prognosis, immune features, and immunotherapy based on immune checkpoint genes
Source: Sci Rep. 2024 Jun 23;14:14431. doi: 10.1038/s41598-024-65198-8 (PMC11194261; doi:10.1038/s41598-024-65198-8)

Supplementary Figure 1: The relationship between ICGs score and clinical characteristics of BLCA patients.


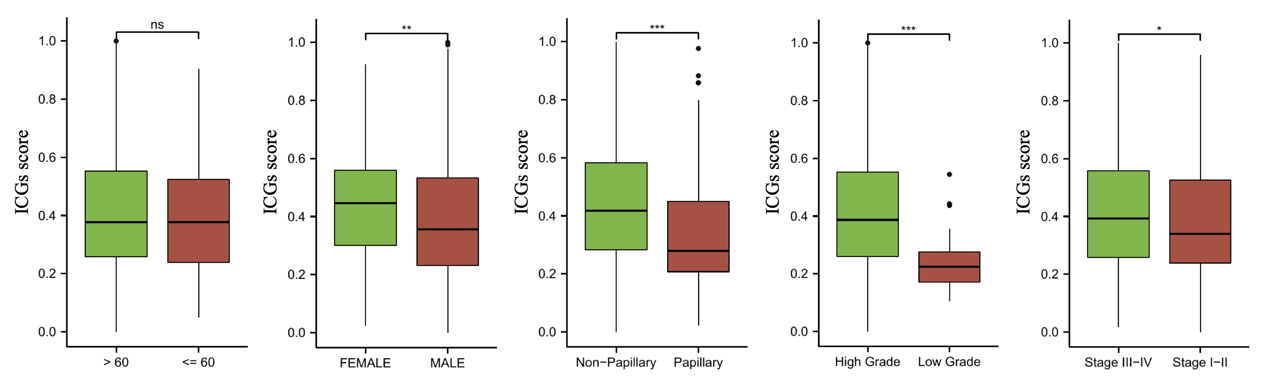

Supplement: Supplementary file 2 — Supplementary Information 2. [file 41598_2024_65198_MOESM2_ESM.docx]
