# Supplementary material for: Identification of bladder cancer subtypes and predictive signature for prognosis, immune features, and immunotherapy based on immune checkpoint genes
Source: Sci Rep. 2024 Jun 23;14:14431. doi: 10.1038/s41598-024-65198-8 (PMC11194261; doi:10.1038/s41598-024-65198-8)

Supplementary Figure 2: (A) The relationships between the molecular subtypes and the different clinical features. (B) The boxplot displaying the difference in immune checkpoint genes between different clusters. The comparison of immunophenoscore (IPS) between different clusters, (C) CTLA4−_PD1−, (D) CTLA4−_PD1+, (E) CTLA4+_PD1−, and (F) CTLA4+_PD1+.


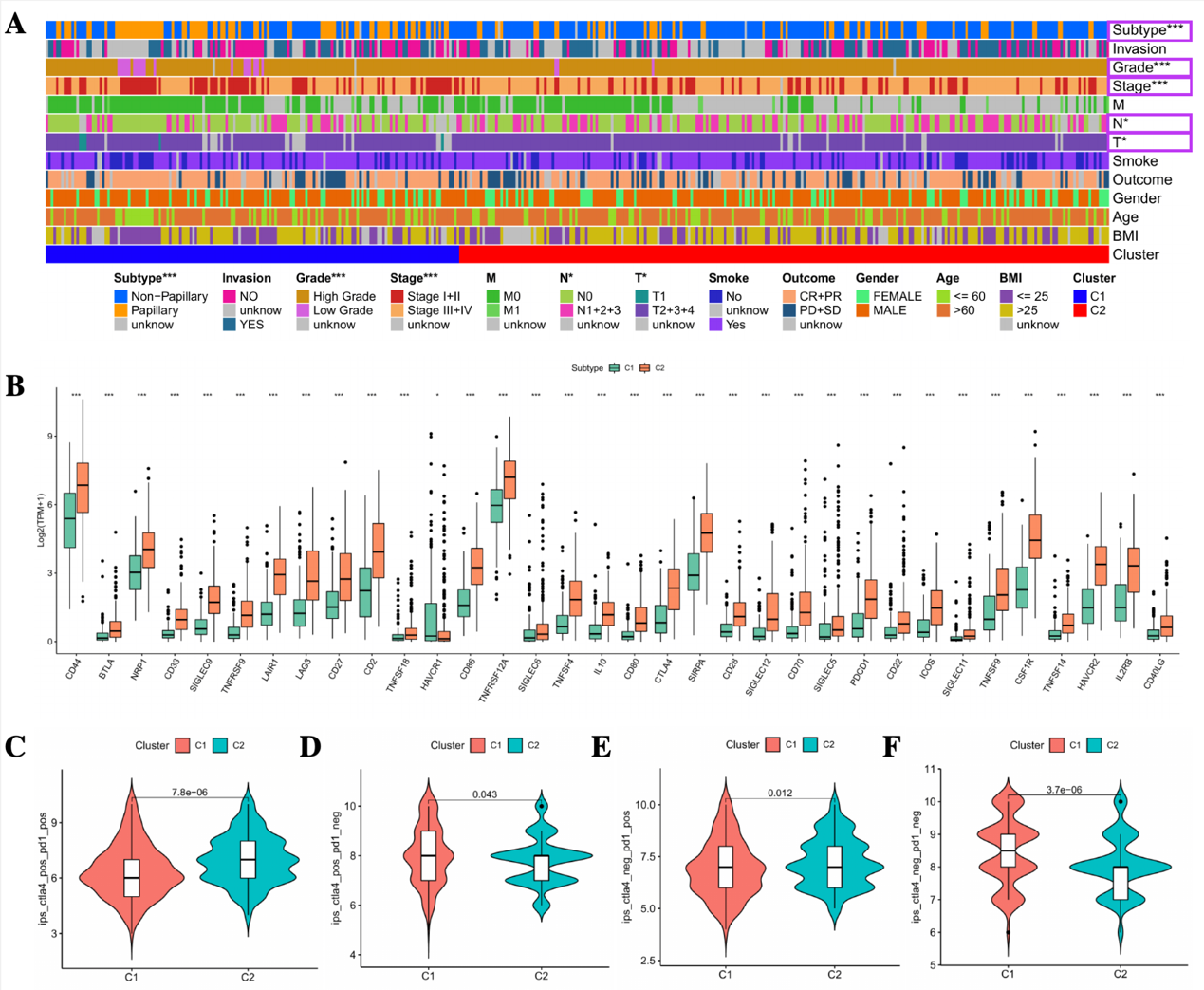

Supplement: Supplementary file 3 — Supplementary Information 3. [file 41598_2024_65198_MOESM3_ESM.docx]
