# Supplementary material for: Identification of bladder cancer subtypes and predictive signature for prognosis, immune features, and immunotherapy based on immune checkpoint genes
Source: Sci Rep. 2024 Jun 23;14:14431. doi: 10.1038/s41598-024-65198-8 (PMC11194261; doi:10.1038/s41598-024-65198-8)

Supplementary Figure 3: The Kaplan–Meier survival analysis showing different survival outcomes for patients grouped by different clinical characteristics in the TCGA-BLCA dataset. (A) Age subgroup; (B) Gender subgroup; (C) BMI subgroup; (D) Clinical stage subgroup.


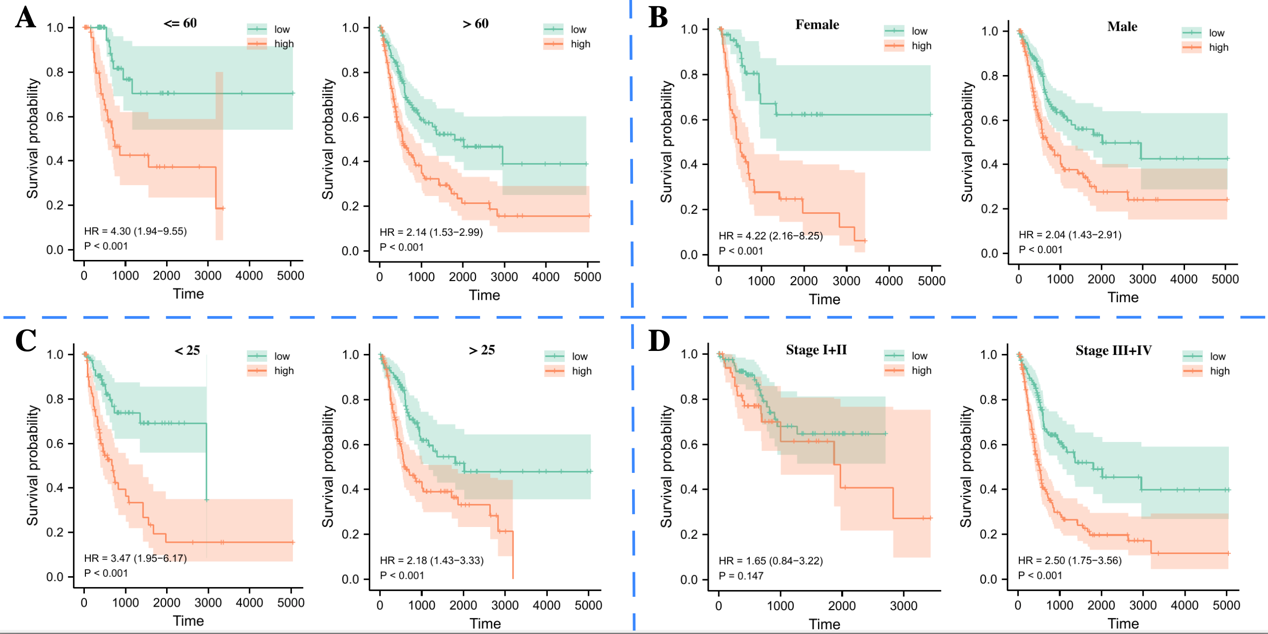

Supplement: Supplementary file 4 — Supplementary Information 4. [file 41598_2024_65198_MOESM4_ESM.docx]
