# Supplementary material for: Identification of bladder cancer subtypes and predictive signature for prognosis, immune features, and immunotherapy based on immune checkpoint genes
Source: Sci Rep. 2024 Jun 23;14:14431. doi: 10.1038/s41598-024-65198-8 (PMC11194261; doi:10.1038/s41598-024-65198-8)

Supplementary Figure 5: The C-index (A), ROC curves (B) and DCA curve curves showing the comparisons of the predictive ability of the risk score, age and tunmor clinical stage.
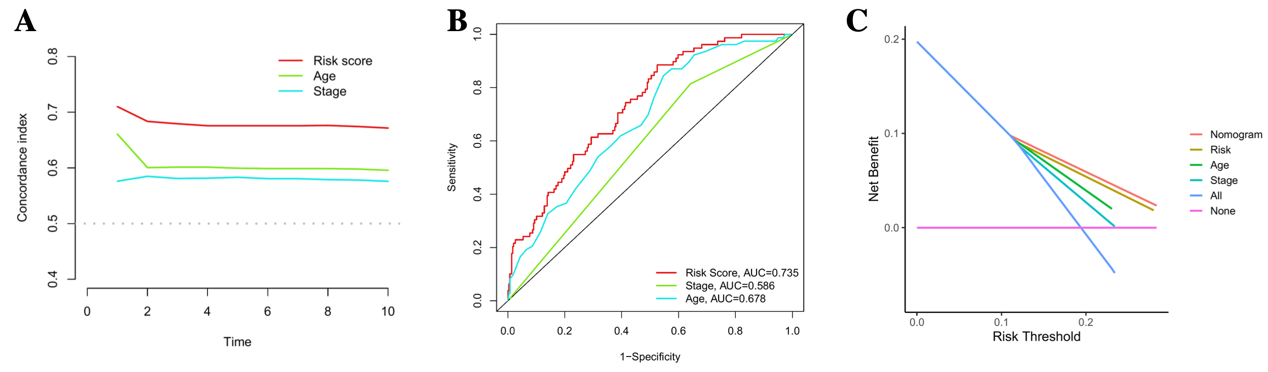

Supplement: Supplementary file 6 — Supplementary Information 6. [file 41598_2024_65198_MOESM6_ESM.docx]
